# Effect of LDL Extracted from Human Plasma on Membrane Stiffness in Living Endothelial Cells and Macrophages via Scanning Ion Conductance Microscopy

**DOI:** 10.3390/cells13040358

**Published:** 2024-02-18

**Authors:** Diana Kiseleva, Vasilii Kolmogorov, Vadim Cherednichenko, Ulyana Khovantseva, Anastasia Bogatyreva, Yuliya Markina, Petr Gorelkin, Alexander Erofeev, Alexander Markin

**Affiliations:** 1 Department of Biophysics, Faculty of Biology, Lomonosov Moscow State University, 119991 Moscow, Russia; 2 Petrovsky National Research Center of Surgery, 119991 Moscow, Russia; vadik2222111@gmail.com (V.C.); nastya.bogatyreva.96@mail.ru (A.B.); yu.v.markina@gmail.com (Y.M.);; 3 Laboratory of Biophysics, National University of Science and Technology MISIS, Leninskiy Prospect, 4, 119049 Moscow, Russia; 4 Medical Institute, Peoples’ Friendship University of Russia named after Patrice Lumumba (RUDN University), 117198 Moscow, Russia

**Keywords:** atherosclerosis, LDL, endothelial cells, intima, scanning ion-conductance microscopy

## Abstract

Mechanical properties of living cells play a crucial role in a wide range of biological functions and pathologies, including atherosclerosis. We used low-stress Scanning Ion-Conductance Microscopy (SICM) correlated with confocal imaging and demonstrated the topographical changes and mechanical properties alterations in EA.hy926 and THP-1 exposed to LDL extracted from CVD patients’ blood samples. We show that the cells stiffened in the presence of LDL, which also triggered caveolae formation. Endothelial cells accumulated less cholesterol in the form of lipid droplets in comparison to THP-1 cells based on fluorescence intensity data and biochemical analysis; however, the effect on Young’s modulus is higher. The cell stiffness is closely connected to the distribution of lipid droplets along the z-axis. In conclusion, we show that the sensitivity of endothelial cells to LDL is higher compared to that of THP-1, triggering changes in the cytoskeleton and membrane stiffness which may result in the increased permeability of the intima layer due to loss of intercellular connections and adhesion.

## 1. Introduction

Atherosclerosis is a chronic inflammation process which affects the complex interaction of various cell types of the vascular wall, blood components, as well as lipid metabolism in the body. Despite many decades of research, it has not been possible to identify any specific causes of the disease itself. Various theories explaining the process of the first fibrous cap development and the atherosclerotic plaque formation rely on several key elements [1]:

1. Disruption of the endothelial layer permeability due to inflammation or in the presence of risk factors such as high blood levels of cholesterol, smoking, diabetes, etcetera.
2. LDL overload and reverse cholesterol transport disorders.
3. Disturbances in the activity of monocytes that enter the vessel wall from the blood and proliferate into macrophages.

The existing theories partially explain the pathogenesis of atherosclerosis from different angles; however, all of them emphasize the key role of the intimal layer covered by the endothelial cells, which are the first to sense stress derived from the intravascular compartment. Any alterations in the intimal layer permeability may become the starting point in the development and progression of the disease. Increased permeability of the intimal layer leads to the LDL and monocytes penetration into the sub-mural wall [2].

Various studies indicate that LDL enters the inner vessel layers in ways other than the cell-to-cell contact disruption and passive transport. Apparently, LDL can be transported via endothelial transcytosis, which represents an active process mediated by several endothelial receptors such as scavenger receptors class B type 1 (SR-B1) and activin-like kinase 1 (ALK1) [3]. Once inside the endothelial cells, cholesterol undergoes hydrolysis in lysosomes and is further esterified in the cytoplasm accumulated in the form of cytoplasmic lipid droplets [4]. The accumulation of cholesterol droplets by cells leads to a transition to mesenchymal state, loss of contacts, and induction of inflammation by neighboring cells (SMCs and macrophages), which also begin to actively accumulate cholesterol. This process completes the formation of foam cells, one of the main morphological components of atherosclerotic plaques. The effect of lipid metabolism can improve the course of atherosclerosis, for example, experiments on mice show that overexpression of LXR, a sterol sensor, in macrophages has an antiatherogenic effect due to an increase in cholesterol efflux [5].

From a biophysical perspective, the process of atherosclerotic plaque formation is characterized by a change in the mechanical properties of endothelial cells, in particular their topography, curvature, and stiffness, since they are the first sensors of external stimuli (shear stress, pressure, and tensile stresses) and extracellular matrix formed by other vessel layers [6]. Studies of the blood flow in atherosclerotic lesion-prone regions have revealed disturbed flow patterns, such as flow separation, recirculation, and reattachment, which may result in longer dwell times for lipoproteins, favoring their permeation into the vessel wall [7].

Many studies demonstrate an increase in arterial wall stiffness in affected patients and as a result of aging [8,9]. However, changes in the stiffness of the entire vessel wall are mostly associated with the loss of elastin fibers and an increase in the number of collagen fibers, as well as with the stiffness of the SMC layer involved in fibrous cap formation in the early stage of atherosclerosis [10,11,12].

In addition, the stiffness of the vascular layers varies at different stages of atherosclerosis at the microscale. Thus, in contrast to the global vessel wall increase in Young’s modulus, measurements of lipid-rich regions in human and ApoE^−/−^ mouse atherosclerotic plaques indicate softening of the intimal layer at the microscale level during diffuse or pathological intimate thickenings [12]. According to the authors, the microscale reduction in Young’s modulus may be explained by the proteoglycans and lipid content increase resulting in void space formation and higher compressibility of the intima and SMCs. Other studies have shown that the plaques found in ApoE^−/−^ mice contain stiffer components including the fibrosis area with increased scarring of connective tissues, calcification, and decreased cellular composition, and the hyper-cellular fibrosis regions with smooth muscle cell hyperproliferation [13]. The lipid-rich foam cell regions showed relatively low Young’s modulus. The authors point out that endothelial cell stiffness may play a major role in plaque stability as well. However, these experiments have been performed on a tissue level instead of a cellular level.

There is a close relationship between the changes in the mechanical properties of the cell and disturbed flow patterns, on the one hand, and intracellular changes, inflammation, and aging, on the other. Changes in the cell mechanical properties play a key role in inducing inflammatory pathways, in particular in the development of atherosclerosis. Cell mechanics are closely related to changes in the cytoskeleton, adhesive function, intercellular interaction [14], and molecular rearrangements. Cellular mechanics can serve both as an independent marker of existing inflammation and as a trigger for similar processes.

Thus, exposure to damaging stimuli results in telomeric and non-telomeric DNA damage, mitochondrial dysfunction, and alterations in energy sensor pathways in endothelial cells [15]. Moreover, senescent endothelial cells change their morphology, become enlarged, flat, and secrete a variety of inflammatory molecules [15].

Internalization of LDL has significant effects on both the plasma membrane and the overall mechanical characteristics of the cell. On the one hand, the cholesterol content can affect the disorganization of membrane rafts, while, on the other hand, it can slow down the processes of transmembrane transport and lead to cytoskeleton restructuring [16,17,18]. In addition, such changes can affect the rate of cell migration [19], which plays an important role in the formation of atherosclerotic lesion.

It is important to emphasize that most studies are conducted on artificially modified oxidized LDL (oxLDL) or aggregated LDL. For example, it was demonstrated that the treatment with oxLDL caused changes in the mechanical properties of endothelial cells by altering the actin cytoskeleton and increasing membrane stiffness [20,21]. Similarly, actin polymerization and defective migration was observed in macrophages incubated with oxLDL [22,23,24].

However, the role of native LDL in influencing vascular cells is still poorly studied. Initially conflicting data on intracellular accumulation of cholesterol during in vitro LDL treatment have led to the hypothesis that oxidative modifications rather than LDL itself play a fundamental role in atherosclerosis progression [25]. However, actual oxLDL values account for a small percentage of total LDL in plasma, with a mean (SD) of 1.27 (0.69) mg/dL and a median (interquartile range) of 1.10 (0.83–1.52) mg/dL, or, as a percentage of LDL, reach a mean (SD) of 1.06 (0.51)% and median (interquartile range) of 0.96 (0.75–1.23)%, although it can reach up to 10% [26]. The same study looked at the aortic pulse wave velocity (aPWV), a marker of arterial stiffness, and its relationship with the level of oxLDL. Their results showed that individuals in the middle oxLDL tertile were equally likely to have a high aPWV compared to individuals in the lowest tertile after adjusting for different CVD risk factors.

Furthermore, with respect to patients with type 2 diabetes, who are known to be more likely to suffer from cardiovascular diseases, oxLDL plaque levels did not predict cardiovascular events [27], highlighting that the presence of oxLDL alone cannot be regarded as an essential factor in the development of atherosclerosis, and in vitro modeling with increased doses of artificially modified LDL may give less clear information about the actual cellular response to plasma LDL.

We decided to conduct our study with the use of native LDL from patients suffering from cardiovascular diseases, without resorting to additional modification. Little is known about the cellular mechanics of professional macrophages, especially the potential effect of native LDL. There are no studies comparing mechanical characteristics of endothelial cells and macrophages or their storage capacity under the same conditions which is the subject of the present study.

In this study, we used SICM for topography and Young’s modulus estimation of living cells under LDL treatment. Previously, we showed that SICM can be successfully used for measuring local mechanical properties of cells [28]. SICM is a technique that uses the intrinsic force between a glass nanopipette tip and a cell membrane [29] to deform the cell surface with known force and estimate a living cell’s Young’s modulus. Intrinsic force could be explained as the electrostatic pressure resulting from a double layer overlap and ion current. Atomic Force Microscopy (AFM) is commonly used to measure cell mechanical properties, while SICM uses less force and pressure on the cell surface. It was demonstrated that SICM can be successfully applied under diverse conditions of alternating cytoskeleton state [30,31]. SICM is additionally combined with confocal microscopy to estimate correlated topography and mechanical properties using confocal imaging, which is crucial to study alteration of mechanical properties and fluorescence labeled LDL accumulation in cells.

We hypothesize that alterations of mechanical properties in endothelial cells due to infiltration and active accumulation of LDL in the subendothelial layer play an important role in the stiffening of the intima and may be involved in the onset and progression of atherosclerosis.

## 2. Materials and Methods

### 2.1. LDL Isolation from Human Plasma

#### 2.1.1. Sample Collection

Low-density lipoproteins were obtained from the blood of 11 patients with cardiovascular diseases, namely coronary heart disease. All patients had chronic heart diseases; patients with acute conditions, as well as systemic autoimmune diseases, were excluded from this study. The general information about the patients, including their medication therapy, comorbidities, and plasma lipid values, is presented in Table S1. All patients provided written informed consent. The study was approved by the Local Ethics Committee of the Petrovsky National Research Center of Surgery (Approval No.3, 17 March 2022). Whole blood samples (30 mL each) were collected into EDTA-tubes and a total volume of approximately 10 mL plasma was obtained from each sample following centrifugation for 20 min at 1600× *g* (3200 rpm on BioSan LMC-4200R, Riga, Latvia).

LDL isolation was performed on the same day as the whole blood samples collection.

#### 2.1.2. Sample Preparation, Centrifugation, Fraction Collection, and Dialysis

Solutions:

- 1.006 g/cm^3^ density solution in Milli-Q water: NaCl 11.42 g/L, EDTA 0.1 g/L
- 1.019 g/cm^3^ density solution prepared in 1.006 g/cm^3^ NaCl/EDTA: KBr 16.5 g/L
- 1.065 g/cm^3^ density solution prepared in 1.006 g/cm^3^ NaCl/EDTA: KBr 77.1 g/L

Isolation protocol:

Crystalline KBr was added to the collected plasma (0.5 g per 1 mL of plasma) and gently vortexed while the salt was completely dissolved (ELMI RM-1L, Riga, Latvia). The saline plasma was transferred to the ultracentrifuge tubes in the ratio of ⅔ of the final volume and ⅓ of top-layer 1.019 g/cm^3^ KBr solution was added. In our study, we used 14 mL of plasma and 7 mL of saline solution. The tubes were carefully balanced in pairs (up to a difference of 0.01 g) and centrifuged in an ultracentrifuge (Beckman L8-55M, Brea, California, USA) for 50 min at 40,000 rpm and +4 °C. The rotor should be cooled in advance (in the refrigerator at +4 °C overnight). After ultracentrifugation, chylomicrons and VLDL should move to the upper fraction, while LDL should remain in the phase separation region. The upper phase should be removed with 1–2 mm left above the phase boundary. A total of 1.065 g/cm^3^ KBr solution should be layered (the required volume depends on the amount of upper fraction that was removed on the previous step: for an initial 21 mL in the tube, we removed approximately 4 mL of chylomicrons and VLDL). After balancing, the tubes were centrifuged for 2 h 10 min at 40,000 rpm and +4 °C. After ultracentrifugation, LDL should move into the upper fraction and form a reddish-brown ring. The LDL ring was carefully assembled. The top third of the top fraction should either be removed in advance or the tip should be dipped at ring level. The LDL fraction was collected with a rotary motion. The collected lipoproteins were dialyzed against 1 L PBS at +4 °C for at least 24 h (day-night-day) with 3–4 buffer changes. After dialysis, LDL was sterilized through a filter with a pore diameter of 0.45 µm. The protein concentration was measured using BioSpec-nano (Shimadzu, Kyoto, Japan). The collected LDL can be stored at +4 °C for two weeks. In our work, we used native LDL; that means that LDL isolated from the serum of patients has not been artificially modified.

### 2.2. Cell Culture, Plating, and Staining Protocol

#### 2.2.1. Cell Culture

THP-1 cells (ATCC, Manassas, Virginia, USA) were grown in RPMI-1640 (Gibco, Thermo Fisher Scientific, Waltham, Massachusetts, USA) and supplemented with 10% FBS (STEMCELL, Cambridge, UK), Penicillin–Streptomycin (STEMCELL, Cambridge, UK), and 3.6 μL 2-Mercaptoethanol (Merck, Darmstadt, Germany) in a 5% CO_2_ humidified atmosphere at 37 °C.

EA.hy926 cells (ATCC, Manassas, Virginia, USA) were grown in DMEM (Gibco, Thermo Fisher Scientific, Waltham, Massachusetts, USA) under the same conditions, except for supplementation with 2-Mercaptoethanol.

#### 2.2.2. Cell Plating

THP-1 cells were seeded on 3.5 cm confocal dishes (BDD-002-035, JetBiofil, Guangzhou, China) in RPMI-1640+10% FBS, with 500–550 thousands cells in 1.5 mL of medium per dish to achieve a final confluence of 70%. THP-1 cells were differentiated with 200 ng/mL phorbol-12-myristate 13-acetate (Thermo Fisher Scientific, Waltham, Massachusetts, USA) for 2 days. Cell differentiation was verified by evaluating cell adhesion and spreading under an optical microscope. On the third day, the control dishes were washed three times with PBS solution and then incubated for 24 h with RPMI-1640 (without FBS and 2-Mercaptoethanol, with Penicillin–Streptomycin). The test dishes were incubated under the same conditions except for the medium containing 100 μg/mL of previously extracted LDL. RPMI without FBS and 2-Mercaptoethanol was used to eliminate additional factors that might provoke lipid droplet accumulation by cells.

EA.hy926 cells were seeded on 3.5 cm confocal dishes (BDD-002-035, JetBiofil, Guangzhou, China) in DMEM + 10% FBS for 24 h, with 250–300 thousands cells in 1.5 mL of medium per dish. After a 24 h incubation and cell adhesion verification, the control and test dishes were prepared as for THP-1, and the cells were incubated in DMEM without FBS (with Penicillin–Streptomycin).

#### 2.2.3. Cell Staining

A stock solution of BDP 630/650 (5 mM) was prepared by dissolving the dye (1.3 mg) in DMSO (1 mL). From this stock solution, a sample with a volume of 1.7 µL was taken and diluted in PBS (9 mL).

All cell lines were stained with BDP 630/650 (Lumiprobe, Moscow, Russia) without fixation for 20 min at 37 °C in the dark, followed by six washes with Hank’s solution.

### 2.3. Biochemical Analysis of Cholesterol Accumulation

The plating protocol for THP-1 and EA.hy926 cells was similar to Section 2.2.2. (Materials and Methods). Cells were seeded in 48-well culture plates (#32048, SPL Life Sciences, Pocheon-si, South Korea), with 500 thousands cells per well.

After a 24 h incubation with LDL, lipids were extracted from the cells using a modified Folch method [32]. The cholesterol detection assay was carried out after extraction using the enzymatic colorimetric method with the CHOLESTEROL liquicolor (HUMAN, Wiesbaden, Germany), according to the manufacturer’s specifications. Taking into account the difference in the division rate between the cell lines, the cholesterol content was adjusted by the total level of protein in a well measured using the Lowry method [33]. The obtained cholesterol-to-protein ratio was normalized by the average value of the control cells within the cell line.

### 2.4. Correlative Scanning Ion-Conductance Microscopy and Confocal Microscopy

SICM, manufactured by ICAPPIC (ICAPPIC ltd, London, UK), was used for topography and Young’s modulus mapping of SH-SY5Y cells. Nanopipettes with a radius of 45–50 nm were made from borosilicate glass O.D. 1.0 mm, I.D. 0.5 mm (WPI, London, UK) by using the laser puller P-2000 (Sutter Instruments, Novato, California, USA). The nanopipette radius was calculated using the following model [34]:

(1) $$r=\frac{Io}{\pi Vktg\left(\alpha \right)}$$

where the half-cone angle α is 3 degrees, k is 1.35 S m^−1^, and V is the applied electrical potential of 200 mV.

The scanning procedure was performed by using the “hopping” mode [35], with a fall rate of 100 nm/ms for the nanopipette and a resolution of 312 nm. The amplitude of the pre-scan was 7000 nm and that of the minimal scan was 700 nm.

A nanopipette was brought up close to the surface and scanned until the ion current through the tip decreased by 2% from its starting value in order to estimate the Young’s modulus of living cells [28]. Following a decrease of 0.5% in the ion current, a noncontact topographic image was obtained. Two more coordinates were obtained at ion current decreases (or setpoints) of 1% and 2%, respectively, corresponding to membrane deformations induced by intrinsic force at each setpoint. Next, Young’s modulus was calculated using the model shown below:

(2) $$E=PA{\left(\frac{Ssub}{Scell}-1\right)}^{-1}$$

where *E* is the estimated Young’s modulus, *P* is the applied pressure, A is a constant depending on the nanopipette geometry, and S_sub_ and S_cell_ are the slopes of the current–distance curve observed between the ion current decreases of 1% and 2% at the non-deformable surface (S_sub_—substrate) and cell surface (S_cell_), respectively. The value of P was obtained from the force–pressure calibration curve [28]. The schematic representation of the current drop comparison is shown in the Supplementary Materials. Data processing was performed using HPICM ImageViewers (ICAPPIC Ltd., London, UK).

Cells were washed several times with Hank’s solution (Gibco, Thermo Fisher Scientific, Waltham, MA, USA) before the scanning procedure. In order to obtain correlative confocal microscopy images of lipid droplets, a 25.3 mW laser beam with a wavelength of 635 nm was focused on the nanopipette tip so that the focal plane of the laser beam and nanopipette tip were in the same place (Figure S1). Then, correlative imaging was performed for the same THP-1 or EA.hy926 cell in Hank’s solution for 1 h per dish. The total number of measured cells for mechanical properties statistics amounted to more than 30 cells in each group. For fluorescence intensity, the number of observations for each sample was three cells per group.

## 3. Results

**Figure 1:**
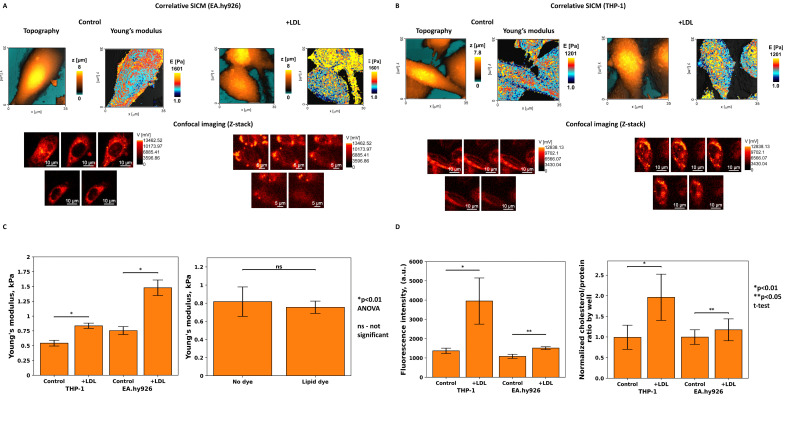
(**A**) Mechanical properties of endothelial cell line EA.hy926 in control plates and LDL treated plates with confocal images of lipid droplets accumulation; (**B**) mechanical properties of PMA-induced THP-1 macrophages in control plates and LDL treated plates with confocal images of lipid droplets accumulation; (**C**) statistical analysis of the difference in Young’s modulus and the effect of BDP 630/650 on membrane stiffness. The small decrease in the stiffness of the endothelial cell membrane caused by the lipid dye is not statistically significant; (**D**) fluorescence intensity measured by correlative SICM and normalized cholesterol-to-protein ratio for EA.hy926 and PMA-induced THP-1 macrophages in control plates andLDL treated plates.

We have produced topographic and quantitative nanomechanical mapping of the THP-1 and EA.hy926 cells before and after incubation with LDL. Correlative imaging (Figure 1A,B) shows major changes in cell topography, Young’s modulus, and cholesterol accumulation on confocal images.

SICM topographic data showed a decrease in the height of EA.hy926 cells after incubation with LDL, as they became more spread out while accumulating cholesterol. There was no significant difference in THP-1 cells in terms of height; however, the accumulation of lipid droplets led to a moderate volume increase. The plasma membrane of both cell lines in the control plate was smoother, whereas LDL exposure stimulated caveolae production.

The five z-stack correlative confocal images reveal areas with lipid droplet accumulation on several levels. Cells with lipid droplets stored from the part adhered to the highest level along the z-axis showed a dramatic increase in membrane stiffness relative to cells with less free cholesterol located only in the lower levels closer to the adhered part. We also noticed a higher cell division rate after LDL incubation; therefore, the variation in cell height, volume, and even quantity of lipid droplets may be explained by discrepancies in the cell cycle.

Both cell lines demonstrated a statistically significant increase in fluorescence intensity; however, the effect size in terms of cholesterol accumulation was greater for THP-1 (Figure 1D). The cholesterol accumulation was also confirmed by enzymatic colorimetric method (Figure 1D), the results aligning with the data obtained from confocal images analysis. Descriptive statistics of cell mechanics, cholesterol accumulation measured by biochemical method, and fluorescence intensity are presented in Table 1.

Despite the lower fluorescence intensity and cholesterol accumulation, the endothelial cell mechanics changed drastically. With respect to EA.hy926, the difference in Young’s modulus between control plates and cells exposed to LDL for 24 h was greater than that for THP-1 cells (Figure 1C and Table 1). Statistical analysis was conducted using ANOVA and Tukey’s HSD test for multiple comparisons when comparing both cell lines and unequal variance t-test for comparison only between control and LDL groups.

We checked the effect of lipid dye on membrane stiffness and topography and confirmed that BDP 630/650 had no statistically significant influence on cellular mechanical characteristics, only leading to a small decrease in stiffness (Figure 1C).

## 4. Discussion

This study was the first to examine the mechanics of living macrophages and endothelial cells exposed to native LDL obtained from patients with cardiovascular disease and their storage capacity. Correlative scanning ion-conductance microscopy allows to measure fluorescence intensity, layer-by-layer distribution of lipid droplets, and mechanical changes from the same living cell.

In this study, we examined the mechanical properties of the endothelial cell line EA.hy926 compared with the macrophage-induced THP-1 as control cells. We found that endothelial cells accumulated cholesterol in the form of lipid droplets in the cytoplasm in the same way as THP-1 cells according to correlative confocal z-stacks. However, the fluorescence intensity highlighted that THP-1 cells, as professional macrophages, accumulate more cholesterol. Cholesterol accumulation, measured using the enzymatic colorimetric method adjusted for protein levels, showed similar results. At the same time, although the changes in membrane stiffness of THP-1 cells were statistically significant, the effect size was not as large as that for endothelial cells.

In another study, the authors studied bone-marrow-derived macrophages isolated from femurs of C57/BL6 mice and used LDL isolated from blood of healthy individuals, which was then oxidized using CuSO_4_ [36]. Mechanical changes were analyzed indirectly, with staining of the actin cytoskeleton with Phalloidin Alexa Fluor-546, and the cells were fixed. Native LDL from healthy donors served as a negative control and actin fluorescence did not change in comparison with control cells without LDL treatment. Oxidized LDL led to increased fluorescence of the cytoskeleton, which may indicate an increase in cell stiffness due to actin polymerization. Actin cytoskeleton rearrangement led to reduced migration ability and formation of lamellipodia.

A previous study conducted on THP-1 cells used native LDL from healthy donors as a base for oxLDL (modified with CuSO_4_), as well as aggregated LDL obtained by vortexing [37]. The working concentration of LDL was similar to that in our work (100 μg/mL). To visualize the accumulation of cholesterol, the authors used Oil Red O. In contrast to our work, accumulation was assessed using gas chromatography–mass spectrometry. The authors found that aggregated LDL caused a significant increase in total free and esterified cholesterol, while oxidized LDL did not cause an increase in either free or esterified cholesterol. Interestingly, incubation with native LDL significantly increased the cellular levels of free cholesterol relative to the control (cells in the medium).

Quantification and visualization of LDL uptake of the human monocytic cell line THP-1 in small concentration (5 μg/mL) was also performed by assessing diI-oxLDL fluorescence by flow cytometry, with the culture treated for 5 days with commercially purchased oxidized low-density lipoproteins [38]. Additionally, to induce the inflammatory pathway and differentiate cells into M1 macrophages, THP-1 cells were treated with LPS and IFN-γ, and, to obtain M2 macrophages, the authors used interleukins 4 and 17. The highest mean fluorescence intensity was obtained for M1 compared to M0 and M2 macrophages. In our work, PMA treatment induced M0 macrophages, and after 24 h of incubation with native LDL the cells demonstrated a high accumulation of cholesterol based on BDP 630/650 fluorescence intensity and enzymatic analysis. In their research, the authors do not demonstrate the dynamics of accumulation at several time points and do not assess cell viability given a prolonged time of LDL treatment.

Lipid accumulation and the number of pseudopodia in macrophages derived from THP-1 monocytes over-expressing CD14 was assessed in another study, where the cells were treated with LDL(−) and in-vitro-modified LDLs (oxidized, aggregated, and acetylated) [39]. Plasma samples were taken from healthy normolipemic subjects, LDL was separated in native LDL (LDL(+) and LDL(−) by anion exchange chromatography), acetylated LDL was prepared by sequential additions of acetic anhydride, and oxidized LDL by incubation with CuSO_4_. HDL was also added to the cells, but did not revert the induction of intracellular lipid accumulation promoted by LDL(−). Morphological changes were observed for both LDL(+) and LDL(−) on macrophages compared to control cells, although the largest external changes were obtained for LDL(−). The uptake of LDL(−) was twice as high as that of LDL(+); furthermore, LDL(−) induced the accumulation of triglyceride even when compared to aggregated LDL.

It is worth noting that triglycerides are carried by very low density lipoproteins (VLDLs) and chylomicrons and are also considered active participants in the development of atherosclerosis. In their study, Deng et al. used VLDL- and chylomicron-sized triglyceride-rich emulsion particles and added them to RAW 264.7 macrophages and human primary macrophages, leading to a marked lipid accumulation [40]. The authors noted that this process was driven by caveolae-mediated endocytosis. In our study, we also noted the formation of caveolae in the cells.

The procedure for isolating LDL from plasma through sequential centrifugation does not imply the homogeneity of the resulting LDL, and the effects observed in studies with native or artificially modified LDL obtained from human blood plasma may also be associated with the presence of proteins related to VLDL or chylomicrons.

No studies were found that assess the macrophage mechanics using a direct method after LDL treatment and, although in professional phagocytes, due to their main function, changes in the cytoskeleton should be flexible depending on external conditions, endothelial cells should not normally demonstrate the same accumulation as macrophages. In this regard, endothelial cells may be more sensitive to the internalization of LDL and our results confirm this assumption. Despite the lower accumulation of native LDL relative to THP-1 cells assessed using the enzymatic method and fluorescence intensity, EA.hy926 cells showed a two-fold increase in membrane stiffness compared to the control.

Several studies have investigated the effect of oxLDL on endothelial cells. Aguilar et al. show that oxLDL caused an increase in membrane stiffness, the formation of lipid rafts on the membrane, an increase in endothelial force generation, and network formation. Particular attention was paid to membrane cholesterol enrichment. They concluded that oxLDL was responsible for biomechanical effects similar to cholesterol depletion rather than cholesterol enrichment, resulting in the disruption of the lipid order [11].

Interesting results have been obtained in a recent study with human aortic endothelial cells treated with native and lipoxygenase oxidized LDL from human plasma, with the cells treated with different concentrations of 50 mg/dL and 250 mg/dL for 24 h [41]. Measurement of total cholesterol in the cells was carried out using the Amplex Red cholesterol assay kit. It was discovered that, at high concentrations, native LDL induced significant cholesterol loading of cells. Using Atomic Force Microscopy, the researchers demonstrated that high doses of native LDL resulted in endothelial stiffening similar to the oxLDL effect, but the effect of native LDL was modest: ~20% increase in elastic modulus between cells exposed to 250 mg/dl versus 50 mg/dl compared to a more than two-fold increase in elastic modulus in cells exposed to oxLDL [21,41,42]. In our study, native LDL caused a change in Young’s modulus, and we used 100 μg/mL which corresponds to the volume of oxLDL used previously [36,37]. In addition, the study does not include information about blood samples.

Human umbilical artery endothelial cells were isolated from the arteries of normal umbilical cords by digestion with collagenase II and incubated with 100 µg/mL of native LDL and oxLDL (oxidized with CuSO_4_) for 3 h. Increasing the time of incubation to 24 h did not affect cell viability; viability decreased with incubation times ≥ 48 h [43]. Catar et al. demonstrated accumulation by visualization with fluorescence microscopy using DiI-labeled particles. Two filters, namely 450 nm and 490 nm, were used. However, the authors presented only one image for the 490 nm filter showing significant accumulation by native LDL without comparison to oxLDL, so it is difficult to assess the real difference in the cholesterol accumulation. At the same time, the authors note that the cells were actively accumulating under native LDL, though less when incubated with oxLDL.

Couto et al. also used the blood of healthy individuals and oxidized LDL modified by CuSO_4_ [44]. Changes in the mechanical properties were assessed using the fluorescence intensity of Alexa 546-conjugated phalloidin for the actin cytoskeleton, as well as by using defocusing microscopy (optical microscopy) where cell stiffness was judged by relaxation time. Upon oxLDL treatment, cells demonstrated a significant increase in actin fluorescence intensity and an increase in cell stiffness, shown by the increase in relaxation time. However, due to the limitation of half the excitation wavelength, optical techniques cannot be used to explore the relationship between cell topography and mechanical properties at the nanoscale level. Furthermore, due to the small size of the nanopipette tip (45–50 nm radius), Young’s modulus measurements can be highly localized to different areas of the living cell. The only disadvantage of SICM may be the low value of deformation (100–200 nm) that does not allow to study properties of intracellular structures.

We also observed a cell stiffness increase and a higher Young’s modulus for areas with excessive cholesterol accumulation along the z-axis. The sensitivity of endothelial cells to LDL was higher compared to THP-1 cells, a phenomenon which may indicate that even small amounts of LDL might trigger severe changes in cytoskeleton, cell adhesion, and intercellular connections in the intimal layer. Moreover, we noticed changes in the plasma membrane surface topography, with caveolae formation as opposed to the smooth surface of control endothelial cells. Caveolae expression in endothelial cells can occur under various stress conditions, for example after 24 hours of shear stress exposure regulating cell membrane tension to adapt to mechanical stress [45]. AFM data on endothelial cells isolated from wild type and Caveolin-1 knockout mice and exposed to oxLDL stress demonstrate that a loss of Caveolin-1 abrogates the uptake of oxLDL and oxLDL-induced endothelial stiffening. In knockout mice, membrane stiffness has been found to decrease when exposed to oxLDL under static and hemodynamic conditions in vitro, as well as in in vivo aortic endothelium from mouse models of dyslipidemia and aging [46].

In general, in the various mentioned works, different concentrations of CuSO_4_ are used for the oxidation of LDL. Moreover, LDL is taken from the plasma of healthy donors, and the atherogenicity, as well as the accumulation of their oxidized modifications by cells, does not always show a clear advantage over aggregation or treatment with native LDL, suggesting that other factors may influence the atherogenicity of LDL. This issue requires additional research.

## 5. Conclusions

In conclusion, we studied the cholesterol accumulation of endothelial and monocytic cell cultures (EA.hy926 and THP-1) exposed to LDL isolated from the plasma of patients with CVD, as well as the formation of lipid droplets and the dependence of the topographic and mechanical properties of cells on the accumulation of LDL. LDL was found to increase membrane stiffness for all cells tested.

Endothelial cells showed a higher sensitivity to cholesterol accumulation. This may be due to less flexible mechanics compared to professional macrophages, indicating their important role in the initial stage of atherosclerosis. Moreover, membrane stiffness correlated with the distribution of lipid droplets within the cell. Compared to control samples, we observed active formation of caveolae on the plasma membrane of endothelial cells. To understand the mechanics of the results obtained and fill a gap regarding the atherogenicity of artificially modified and native LDL, it is necessary to conduct additional analyses of LDL samples.

## Figures and Tables

**Table 1:** Descriptive statistics for fluorescence intensity, normalized cholesterol-to-protein ratio, and Young’s modulus for both groups in THP-1 and EA.hy926 cells.

Cell Line	Group	Mean	SD *
Fluorescence intensity			
THP-1	Control	1368.43	140.29
	LDL	3951.08	1198.39
EA.hy926	Control	1083.47	108.84
	LDL	1513.84	70.20
Normalized cholesterol-to-protein ratio			
THP-1	Control	1.00	0.29
	LDL	1.97	0.55
EA.hy926	Control	1.00	0.17
	LDL	1.18	0.26
Young’s modulus, kPa			
THP-1	Control	0.54	0.05
	LDL	0.84	0.05
EA.hy926	Control	0.76	0.07
	LDL	1.48	0.13

* SD—standard deviation.

## Data Availability

The data that support the findings of this study are available from the corresponding author, D. Kiseleva, upon reasonable request.

## Supplementary Materials

The following supporting information can be downloaded at: https://www.mdpi.com/article/10.3390/cells13040358/s1, Figure S1: The schematic illustration of correlative SICM and confocal imaging and the representation of current drop comparison; Table S1: Baseline characteristics of the study population.
